# Osteoblast-derived EGFL6 couples angiogenesis to osteogenesis during bone repair

**DOI:** 10.7150/thno.60902

**Published:** 2021-09-27

**Authors:** Kai Chen, Shijie Liao, Yicheng Li, Haibo Jiang, Yun Liu, Chao Wang, Vincent Kuek, Jacob Kenny, Boxiang Li, Qian Huang, Jianxin Hong, Yan Huang, Shek Man Chim, Jennifer Tickner, Nathan J. Pavlos, Jinmin Zhao, Qian Liu, An Qin, Jiake Xu

**Affiliations:** 1 School of Biomedical Sciences, University of Western Australia, Perth, WA, Australia.; 2 Department of Orthopedics, The First Affiliated Hospital of Wenzhou Medical University, Wenzhou, Zhejiang, China.; 3 Guangxi Key Laboratory of Regenerative Medicine, Guangxi Medical University, Nanning, Guangxi, China.; 4 Department of Orthopedics, The First Affiliated Hospital of Guangxi Medical University, Nanning, Guangxi, China.; 5 School of Molecular Sciences, University of Western Australia, Perth, WA, Australia.; 6 Department of Chemistry, The University of Hong Kong, Pokfulam Road, Hong Kong, P. R. China.; 7 Wenzhou Medical University, Wenzhou, Zhejiang, China.; 8 Shanghai Key Laboratory of Orthopedic Implants, Department of Orthopedics, Shanghai Ninth People's Hospital, Shanghai Jiao Tong University School of Medicine, Shanghai, China.

**Keywords:** angiogenesis, osteogenesis, EGFL6, bone defect, bone repair

## Abstract

**Rationale:** Angiogenesis and osteogenesis are highly coupled processes which are indispensable to bone repair. However, the underlying mechanism(s) remain elusive. To bridge the gap in understanding the coupling process is crucial to develop corresponding solutions to abnormal bone healing. Epidermal growth factor-like protein 6 (EGFL6) is an angiogenic factor specifically and distinctively up-regulated during osteoblast differentiation. In contrast with most currently known osteoblast-derived coupling factors, EGFL6 is highlighted with little or low expression in other cells and tissues.

**Methods:** In this study, primary bone marrow mesenchymal stem cells (MSCs) and osteoblastic cell line (MC3T3-E1) were transduced with lentiviral silencing or overexpression constructs targeting EGFL6. Cells were induced by osteogenic medium, followed by the evaluation of mineralization as well as related gene and protein expression. Global and conditional knockout mice were established to examine the bone phenotype under physiological condition. Furthermore, bone defect models were created to investigate the outcome of bone repair in mice lacking EGFL6 expression.

**Results:** We show that overexpression of EGFL6 markedly enhances osteogenic capacity *in vitro* by augmenting bone morphogenic protein (BMP)-Smad and MAPK signaling, whereas downregulation of EGFL6 diminishes osteoblastic mineralization. Interestingly, osteoblast differentiation was not affected by the exogenous addition of EGFL6 protein, thereby indicating that EGFL6 may regulate osteoblastic function in an intracrine manner. Mice with osteoblast-specific and global knockout of EGFL6 surprisingly exhibit a normal bone phenotype under physiological conditions. However, EGFL6-deficiency leads to compromised bone repair in a bone defect model which is characterized by decreased formation of type H vessels as well as osteoblast lineage cells.

**Conclusions:** Together, these data demonstrate that EGFL6 serves as an essential regulator to couple osteogenesis to angiogenesis during bone repair.

## Introduction

Bone defects often arise as sequelae of incidents such as trauma, infection, surgical resection, or congenital conditions. As a highly regenerative tissue, bone undergoes efficiently repair process following injuries 1. Unfortunately, 5 to 10% patients with fractures are estimated to suffer from delayed union or nonunion 2, 3. Angiogenesis and osteogenesis are considered as two indispensable steps involved in successful bone repair. Type H vessels have recently been identified as bone-specific vessel type characterized by strong positivity for CD31 and endomucin (EMCN) 4. It is well appreciated that this capillary subtype sustains bone homeostasis by coupling angiogenesis to osteogenesis, and compromised CD31^high^EMCN^high^ endothelium leads to impaired osteogenesis 5, 6. However, the nature and identities of regulatory factors that couple angiogenesis to osteogenesis during the process of bone repair within bone microenvironment remain largely unknown.

We have previously demonstrated that epidermal growth factor-like protein 6 (EGFL6) is a novel angiogenic factor prominently expressed during osteoblast differentiation 7. EGFL6 is a secreted protein that promotes endothelial cell (EC) migration as well as angiogenesis via the induction and phosphorylation of ERK 7, suggesting that osteoblast derived EGFL6 may mediate angiogenesis in a paracrine manner within the skeletal system. EGFL6 targets integrins and regulates an array of biological activities through arginine-glycine-aspartic acid (RGD) domain 8, 9. Recently, EGFL6 was shown to interact directly with β1 integrin in ECs as indicated by co-immunoprecipitation 10. Thus, given the essential role of endothelial β1 integrin on the phenotype of bone-specific vessels 11, we hypothesized that EGFL6 may regulate the angiogenesis in the context of the bone microenvironment. On the other hand, although our previous study showed addition of exogenous soluble EGFL6 protein presented lack of effect on the proliferation and differentiation of osteoblasts, it is currently unclear whether and how intracellular expression of EGFL6 influences osteoblast differentiation.

Here, we comprehensively explored the function of EGFL6 in osteoblast lineage cells by investigating the osteogenic capacity of primary bone marrow-derived mesenchymal stem cells (MSCs) and MC3T3-E1 cells with overexpression or depletion of EGFL6. Our findings suggest that EGFL6 positively regulates osteoblast differentiation through an intracrine mechanism. Under physiological conditions, conditional knockout (cKO) of the EGFL6 gene in the osteocalcin (OCN)-expressing lineage cells and global KO (gKO) exhibit no overt bone phenotype. However, when EGF6 cKO mice were subjected to pathological bone defects, bone repair is delayed owing to a reduction in osteoblasts and type H vessels. Thus, our study uncovers EGFL6 as a candidate osteoblast-derived angiogenic factor required for the coupling of osteogenesis to angiogenesis during bone defect repair.

## Results

### EGFL6 is specifically expressed during osteoblast differentiation and co-localizes with blood vessels in bone

**Figure 1:**
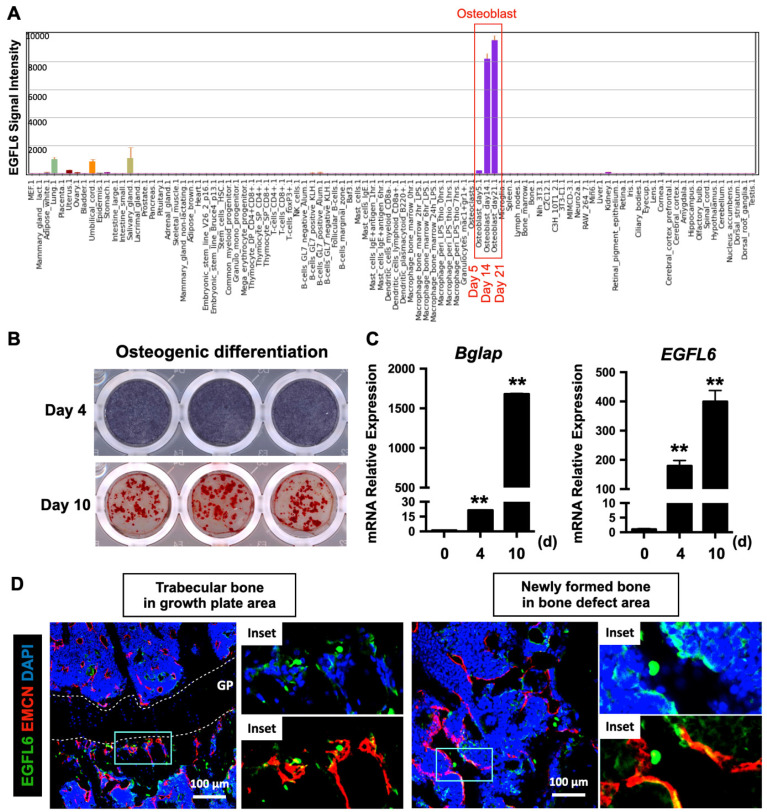
** EGFL6 is highly expressed during osteoblast differentiation and co-localizes with blood vessels in bone. (A)** EGFL6 expression profiled from an array of normal tissues, organs, and cell lines in mice. Data is adapted from BioGPS (http://biogps.org/). Red box indicates the EGFL6 signal intensity in osteoblasts. **(B)** Alkaline phosphatase (ALP) staining and Alizarin Red S (ARS) staining showing the osteogenic differentiation of neonatal calvarial osteoblasts on day 4 and day 10 respectively. **(C)** qPCR analysis of osteogenic gene *Bglap* as well as *EGFL6* during osteoblast differentiation (n = 3 per group). **(D)** Representative confocal images of the immunostaining of EGFL6 and endomucin (EMCN) in 12-week-old male mice tibiae and bone defect area. Growth plate (GP) is indicated with white dashed line. All data are presented as mean ± SD. **P < 0.01 relative to the control group. Differences are analyzed using Student's t-test.

To investigate the transcriptional activity of EGFL6 in different biological samples, a high-throughput gene expression profiling was conducted from an array of normal tissues, organs, and cell line in mice through BioGPS portal (http://biogps.org/) 12, 13 using Mouse MOE430 Gene Atlas (GSE10246) 14. This revealed that EGFL6 is the most prominently expressed during osteoblast differentiation, particularly at the mid-late stage (Figure 1A). To confirm this, neonatal calvarial osteoblasts were isolated and differentiated under osteogenic conditions. Alkaline phosphatase (ALP) staining on day 4 and alizarin red S (ARS) staining on day 10 were used to validate the process of osteoblast differentiation (Figure 1B). Further qPCR analysis showed that the mRNA expression level of EGFL6 dramatically increased as osteoblasts differentiated towards maturity, exhibiting a paralleled expression pattern of osteoblast marker gene - *Bglap* (Figure 1C), a finding consistent with our previous reports 7. Intriguingly, compared with currently known osteoblast-derived angiogenic factors (e.g. EGFL7 15, Slit3 16, Vegfa 17, and Cxcl9 18) which are also considerably expressed in other cells and tissues, EGFL6 is expressed most abundantly and specifically expressed in osteoblasts (Figure S1). Furthermore, we also observed that EGFL6 is highly expressed in trabecular bone adjacent to the growth plate area as well as in sites undergoing active bone repair (Figure 1D). In addition, EGFL6 partly co-localized with the EMCN-positive endothelium (Figure 1D), implying a potential role in angiogenesis within the bone microenvironment. Accordingly, we found that the expression of EGFL6 was strongly upregulated in the bone callus during bone injury healing in a rodent osteotomy model, which exhibited a biphasic expression of EGFL6 that largely mimicked the angiogenic factor VEGF throughout the healing process (Figure S2). Based on these expression and localization data we hypothesized that EGFL6 may function in osteoblasts and endothelial cells as a dual regulator for osteogenesis and angiogenesis during bone repair.

### Intracellular EGFL6 mediates osteoblast differentiation through the BMP-Smad and MAPK signaling pathway

**Figure 2:**
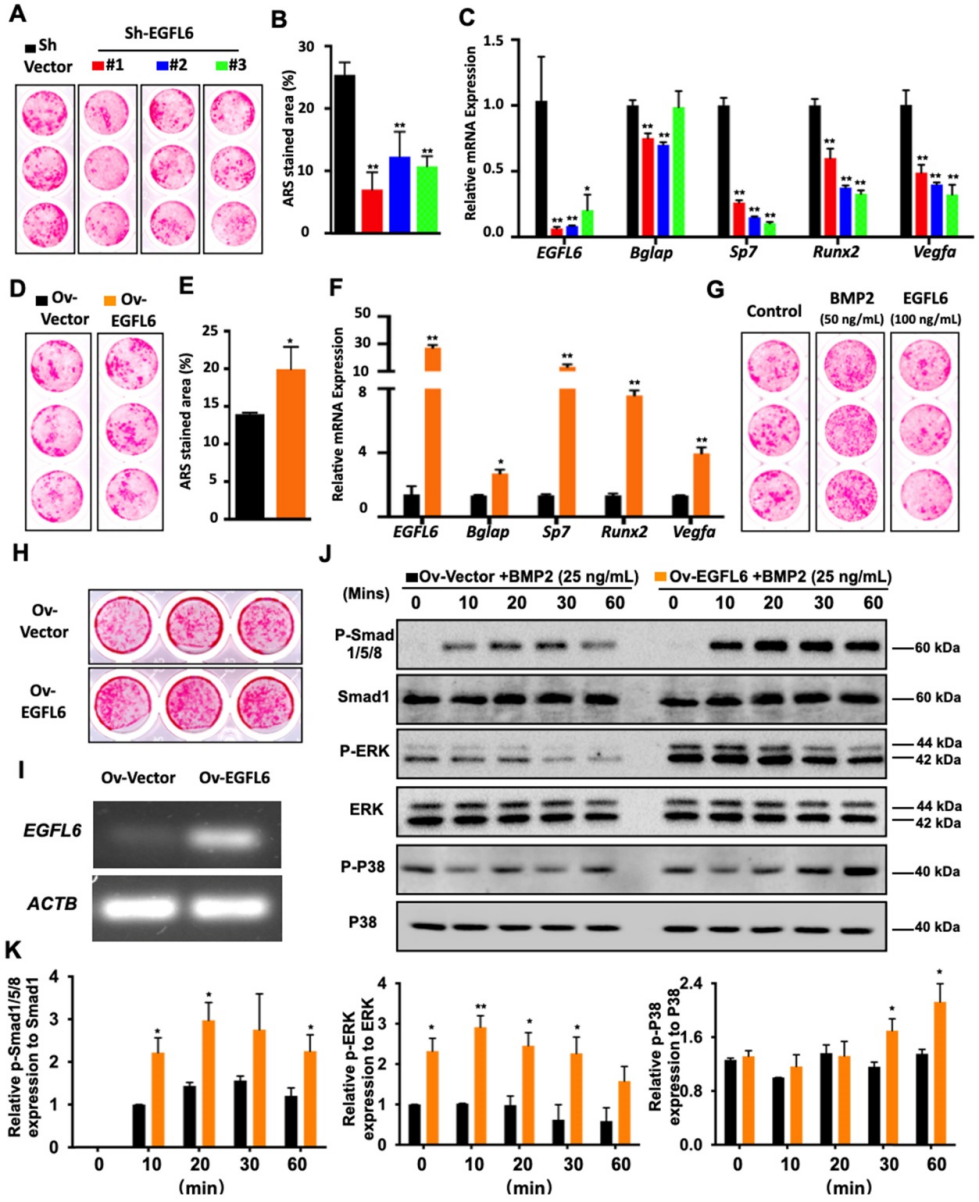
** EGFL6 mediates osteoblast differentiation through BMP-Smad and MAPK signaling pathways. (A)** Alizarin Red S (ARS) staining of mesenchymal stem cells (MSCs) transduced with lentiviral EGFL6 shRNA or a vector control and induced osteogenic differentiation for 14 days. **(B)** Quantification of ARS-stained area of (A) (n = 3 per group). **(C)** qPCR analysis of mRNA expressions of *EGFL6*, *Bglap* (encoding osteocalcin), Sp7 (encoding osterix), *Runx2*, and *Vegfa* (n = 3 per group). **(D)** ARS staining of MSCs transduced with lentiviral EGFL6 overexpression vector or a vector control and induced osteogenic differentiation for 14 days. **(E)** Quantification of ARS-stained area of (D) (n = 3 per group). **(F)** qPCR analysis of mRNA expressions of *EGFL6, Bglap, Sp7, Runx2*, and *Vegfa* (n = 3 per group). **(G)** ARS staining of MSCs induced into mineralization in the absence or presence of BMP2 and EGFL6 protein. **(H)** ARS staining of the mineralization of MC3T3-E1 cells stably transduced with lentiviral EGFL6 overexpressing vector or a control vector (n = 3 per group). **(I)** Gel electrophoresis showing EGFL6 expression in MC3T3-E1 cells following osteogenic induction for 21 days. **(J)** Western Blot assay showing the proteins level of basal canonical (Smad) and non-canonical (MAPK) BMP signaling pathways including P-Smad 1/5/8, P-ERK, and P-P38 in MC3T3-E1 cells induced by BMP-2. **(K)** Quantifications of the band intensities of (J) (n = 3 per group). All bar graphs are presented as mean ± SD. *P < 0.05, **P < 0.01 relative to the control group. Differences are analyzed using Student's t-test.

Because EGFL6 is highly expressed during osteoblast differentiation, we next assessed whether EGFL6 can regulate osteogenesis. Our previous study demonstrated the addition of exogenous soluble EGFL6 had no effect on osteoblasts mineralization 7, but it remains unclear whether the alterations of endogenous EGFL6 levels can regulate osteoblast differentiation via an intracrine mode of action. To this end, we comprehensively investigated whether intracellular or endogenous EGFL6 expression level influences the osteogenic capacity of osteoblast lineage cells. Bone marrow-derived mesenchymal stem cells (MSCs) transduced with three different lentiviral shRNA constructs targeting EGFL6 (shRNA#1, #2, #3) or a vector control (sh-Vector) were used to induce osteogenic differentiation for 14 days. ARS staining showed that the formation of bone nodules was largely reduced following the knockdown of EGFL6 expression (Figure 2A-B). The knockdown efficiency of EGFL6 was verified by qPCR analysis (Figure 2C). Furthermore, an array of osteogenic marker genes including *Bglap* (encoding osteocalcin), *Sp7* (encoding osterix), and *Runx2* were significantly downregulated following the loss of EGFL6, as indicated by the qPCR analysis (Figure 2C). Interestingly, the expression of osteoblast-derived angiogenic factor *Vegfa* was also affected due to the knockdown of EGFL6 (Figure 2C). Conversely, the transduction of lentiviral EGFL6 overexpression leads to an enhanced osteoblast mineralization as indicated by ARS staining (Figure 2D-E). Consistently, the above osteogenic genes as well as *Vegfa* were also significantly up-regulated (Figure 2F). However, MSCs mineralization remained unchanged when exposed to exogenous EGFL6 protein (100 ng/mL) as compared with the control (Figure 2G). Therefore, we reasoned that it is intracellular EGFL6 that regulates osteogenic activity in an intracrine manner. To further explore how EGFL6 mediates osteoblast differentiation, we analyzed basal canonical (Smad) and non-canonical (MAPK) BMP signaling pathways in MC3T3-E1 cells stably transduced with vector control (Ov-Vector) or EGFL6 overexpression (Ov-EGFL6) construct. MC3T3-E1 cells stably overexpressing EGFL6 consistently exhibited asignificant increase in bone nodule formation (Figure 2H-I). In addition, osteoblast-related markers (runx2 and osteocalcin) and VEGF were also found to be up-regulated at the protein levels (Figure S3). Following the BMP2 (25 ng/mL) stimulation, the phosphorylations of Smad1/5/8 as well as ERK and p38 were increased in osteoblasts overexpressing EGFL6 (Figure 2J-K). However, the addition of exogenous soluble EGFL6 protein failed to activate the BMP-MAPK signaling pathway (Figure S4), suggesting that EGFL6 may positively mediate osteogenic differentiation in an intracrine manner.

### Osteoblast-specific deletion of EGFL6 and global knockout of EGFL6 do not alter bone homeostasis under physiological settings

**Figure 3:**
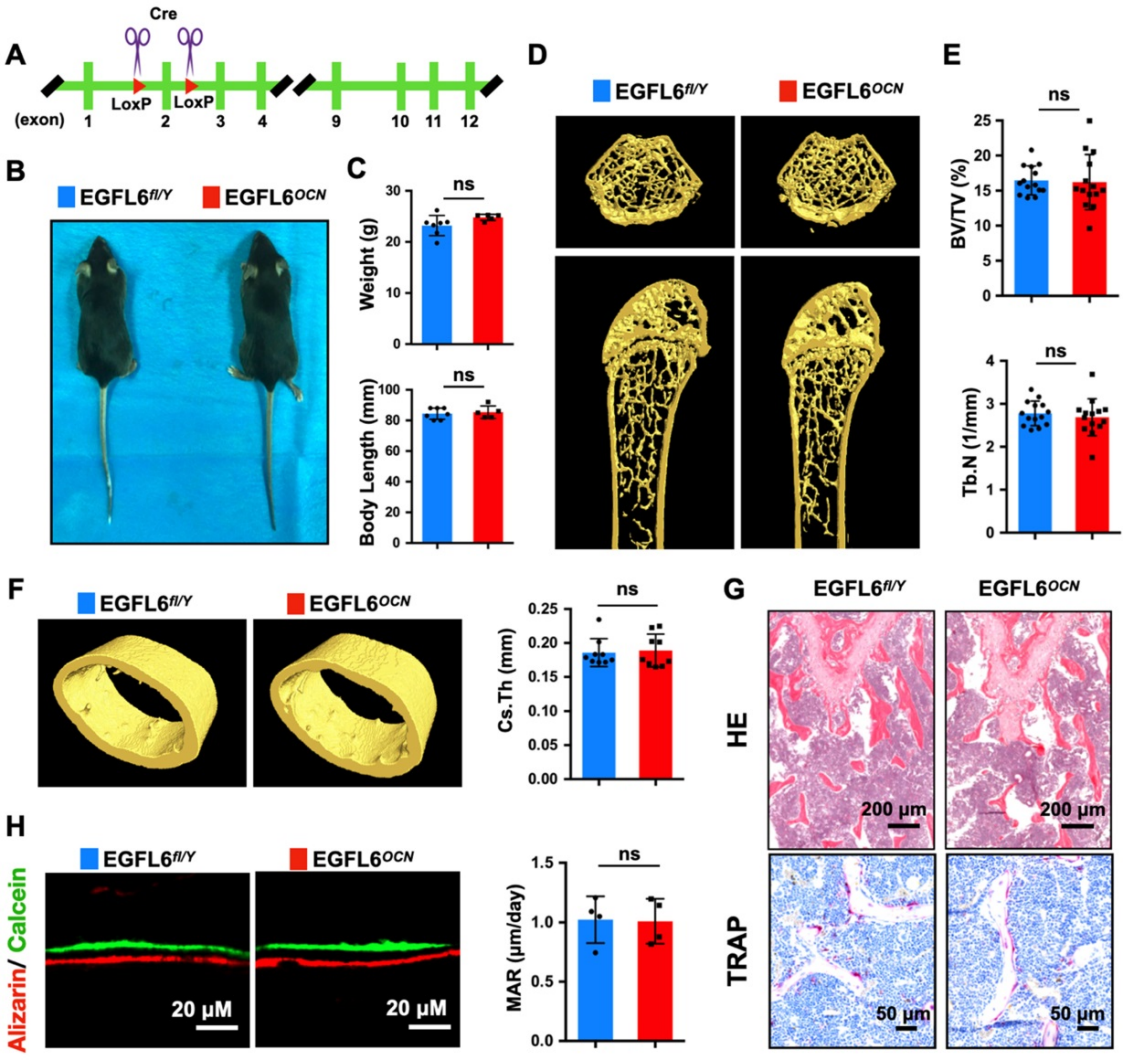
** Osteoblast-specific deletion of EGFL6 has no significant effect on bone phenotype. (A)** Schematic illustration of the strategy used to generate the osteoblast - specific EGFL6 conditional knockout (cKO) mice. **(B)** Photographs of a 12-week-old male EGF6 conditional knockout mouse (EGFL6^OCN^) and its littermate control (EGFL6^fl/Y^). **(C)** Weight and body length of 12-week-old male EGFL6^OCN^ mice (n = 5) and EGFL6^fl/Y^ mice (n = 7). **(D)** Representative three-dimensional reconstructed micro-CT images showing the femurs 12-week-old male EGFL6^OCN^ and EGFL6^fl/Y^ mice. **(E)** Quantification of the trabecular bone parameters including bone volume per tissue volume (BV/TV) and trabecular number (Tb.N) (n = 14 per group). **(F)** Representative micro-CT images of cortical bone, and quantification of cross-sectional thickness (Cs.Th) of the cortical bone (n = 9 per group). **(G)** Representative hematoxylin-eosin (HE) and tartrate-resistant acid phosphatase (TRAP) staining of femurs. **(H)** Representative images of bone growth rates as determined by calcein and alizarin red labelling, and quantification of mineral apposition rate (MAR) (n = 4 per group). All bar graphs are presented as mean ± SD. ns, no significance. Differences are analyzed using Student's t-test.

Based on our findings of EGFL6's role during osteoblast differentiation, we next assessed the potential physiological role of EGFL6 in bone remodeling. For this, osteoblast-specific EGFL6 conditional knockout (cKO) mice (OCN-Cre, EGFL6^ fl/Y^; EGFL6^OCN^ hereafter) were generated by crossing EGFL6-floxed mice (EGFL6^fl/Y^) with osteocalcin (OCN)-cre mice (Figure 3A). Unexpectedly, EGFL6^OCN^ mice (12-week-old, male) exhibited a similar stature and body weight with the EGFL6^fl/Y^ littermate control mice (Figure 3B-C). In addition, micro-computed tomography (micro-CT) scanning and analyses revealed that the bone parameters of trabecular bone (bone volume per tissue volume, BV/TV; trabecular number, Tb.N) as well as the cortical bone (cross-sectional thickness, Cs.Th) were unaltered in the male EGFL6^OCN^ mice as compared with EGFL6^fl/Y^ mice (Figure 3D-F). Consistently, hematoxylin-eosin (HE) and tartrate-resistant acid phosphatase (TRAP) staining of distal femurs sections from 12-week-old EGFL6^OCN^ and EGFL6^fl/Y^ mice exhibited no significant difference in trabecular bone as well as osteoclast activity (Figure 3G). Furthermore, by double fluorescent labelling of calcein and ARS, we found that mineral apposition rate (MAR) of calvarial bone in EGFL6^OCN^ is similar with that of littermate EGFL6^fl/Y^ mice (Figure 3H). Similarly, no overt differences were observed in bones of female cKO mice when compared with wildtype (EGFL6^fl/fl^) mice (Figure S5).

**Figure 4:**
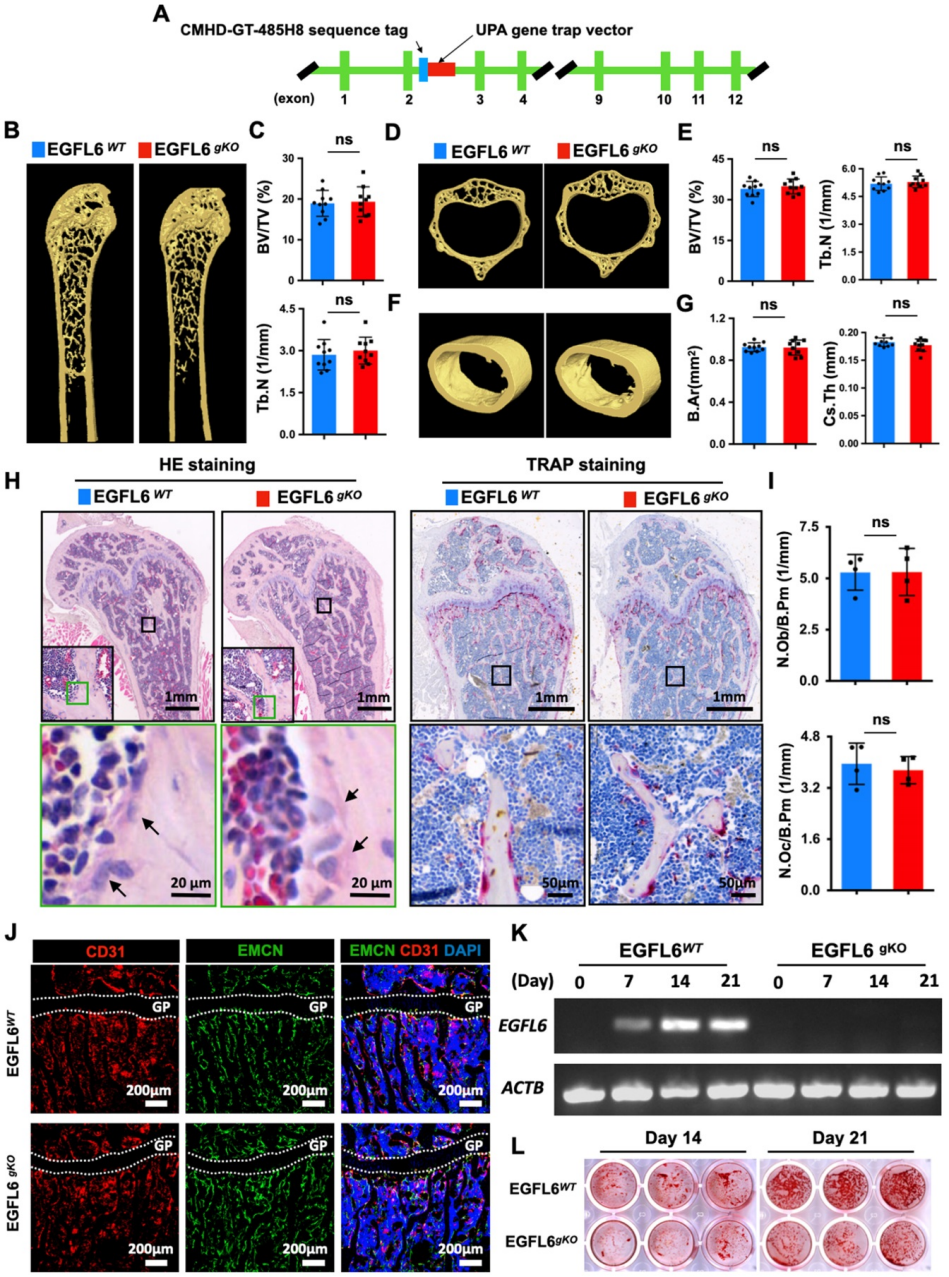
** EGFL6 global knockout (gKO) mice display normal bone phenotype. (A)** Schematic illustration of the strategy used to generate the EGFL6 global knockout (gKO) mice. **(B)** Representative three-dimensional reconstructed micro-CT images showing the femurs of 24-week-old male EGFL6^WT^ and EGFL6^gKO^ mice. **(C)** Quantification of the trabecular bone parameters including bone volume per tissue volume (BV/TV) and trabecular number (Tb.N) (n = 10 per group). **(D)** Representative micro-CT images of lumbar 1 (L1) of EGFL6^WT^ and EGFL6^gKO^ mice, and **(E)** quantification of BV/TV and Tb.N of the trabecular bone (n = 10 per group). **(F)** Representative micro-CT images of cortical bone of EGFL6^WT^ and EGFL6^gKO^ mice, and **(G)** quantification of bone marrow area (B.Ar) and cross-sectional thickness (Cs.Th) of the cortical bone (n = 10 per group). **(H)** Representative hematoxylin-eosin (HE) and tartrate-resistant acid phosphatase (TRAP) staining of femurs of EGFL6^WT^ and EGFL6^gKO^ mice. Black arrows indicate osteoblasts. **(I)** Quantifications of number of osteoblast per bone perimeter (N.Ob/Pm) and number of osteoclast per bone perimeter (N.Oc/Pm). **(J)** Representative confocal images of type H vessel stained for CD31 (red) and EMCN (green) in tibia of EGFL6^WT^ and EGFL6^gKO^ mice. **(K)** EGFL6 gene expression in differentiating bone marrow mesenchymal stem cells (MSCs) derived from EGFL6^WT^ and EGFL6^gKO^ mice. **(L)** Alizarin red S (ARS) staining of mineralization of MSCs from EGFL6^WT^ and EGFL6^gKO^ mice. Growth plate is indicated with white dashed line. All bar graphs are presented as mean ± SD. ns, no significance. Differences are analyzed using Student's t-test.

As bone may not be the exclusive source of EGFL6, we rationalized that EGFL6 from other organs may compensate for the absence of EGFL6 in the bones of EGFL6^ OCN^ mice. To this end, we generated EGFL6 global knockout (gKO) mice by inserting UPA gene trap vector downstream of CMHD-GT-485H8 sequence tag in the EGFL6 genome (Figure 4A). However, Surprisingly, the bone phenotype of EGFL6 gKO mice was indistinguishable from WT littermates as indicated by micro-CT scanning and analysis, including femur trabecular bone (Figure 4B-C), vertebrate trabecular bone (Figure 4D-E), and femur cortical bone (Figure 4F-G). Moreover, bone histomorphometry analysis showed comparable osteoblast as well as osteoclast numbers (Figure 4H-I). Bone cryosections stained by CD31 and EMCN revealed that type H vessels remained unchanged in the EGFL6-deficient mice (Figure 4J). Taken together, these results indicated that loss-of-EGFL6 in osteoblasts and/or peripheral tissues does not influence bone homeostasis under the physiological settings. On the other hand, MSCs isolated from EGFL6 gKO mice presented a compromised bone nodule formation as compared with that from WT mice (Figure 4K-L), a finding that was paralleled by the downregulation of osteoblast-related genes (Figure S6).

### Deletion of EGFL6 in osteoblasts leads to compromised bone repair

**Figure 5:**
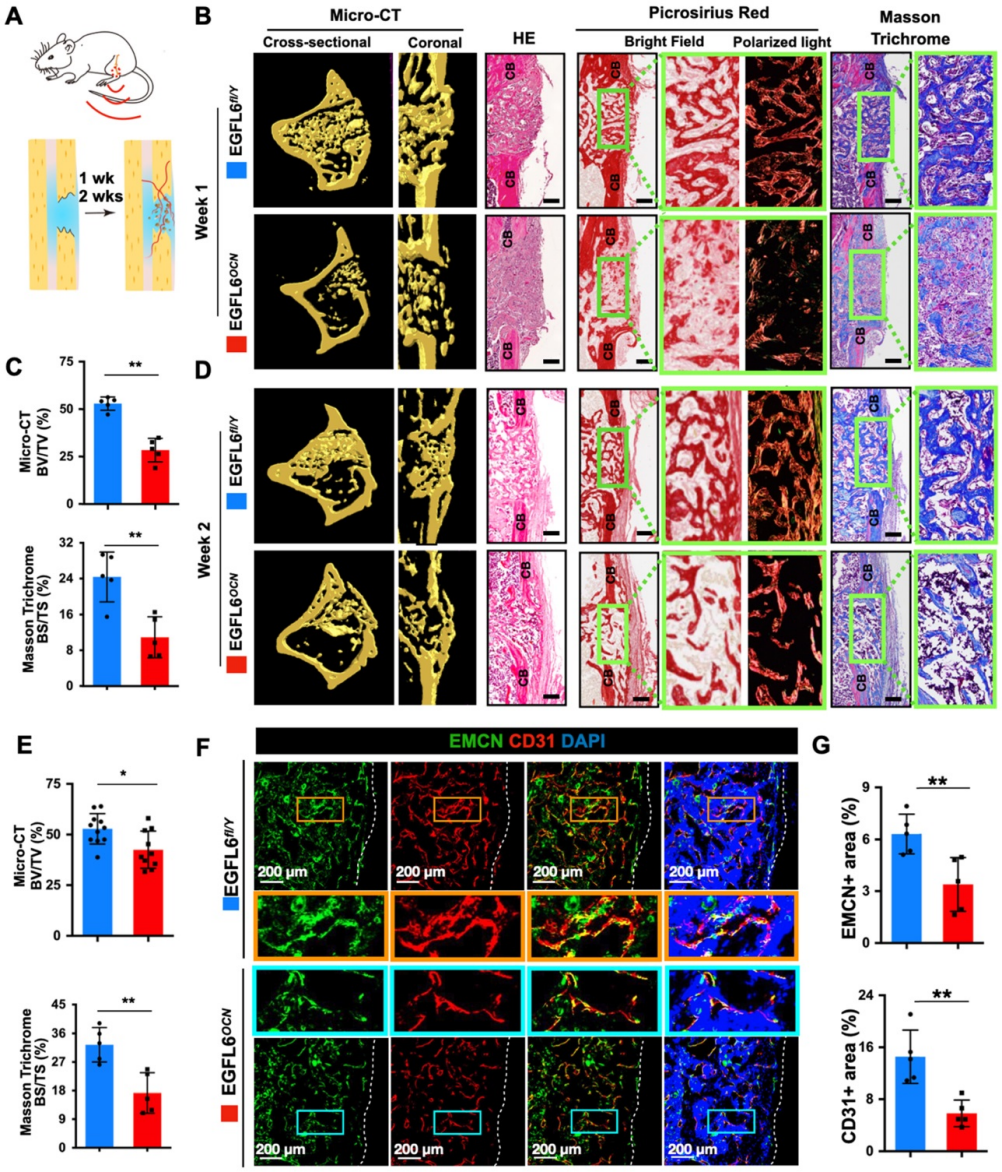
** Deletion of EGFL6 in osteoblasts leads to impaired bone repair characterized by reduced angiogenesis. (A)** Schematic illustration of mono-cortical bone defect model. **(B)** Representative three-dimensional reconstructed micro-CT images of bone repair in EGFL6^OCN^ and EGFL6^fl/Y^ mice 1 week after surgical procedure, and bone histomorphometric analysis including hematoxylin-eosin (HE), picrosirius red (PSR), Masson trichrome staining of bone defect region. CB, cortical bone. Scale bar = 200 μm **(C)** Quantification of newly formed bone in defect region by micro-CT scanning and Masson trichrome staining (n = 5 per group). BV/TV, bone volume per tissue volume; BS/TS, bone surface and tissue surface. **(D)** Representative micro-CT and bone histomorphometric images of bone defect at week 2. Scale bar = 200 μm.**(E)** Quantification of the newly formed bone in defect region at week 2 by micro-CT scaning (WT, n = 11; cKO, n = 10) and Masson trichrome staining (n = 5 per group). **(F)** Representative confocal images of type H vessels stained for CD31 (red) and EMCN (green) in bone defect region of EGFL6^OCN^ and EGFL6^fl/Y^ mice at week 2. **(G)** Quantification of EMCN^+^ and CD31^+^ area in (F) (n = 5 per group). White dashed lines indicate the edge of bone tissue. All bar graphs are presented as mean ± SD. *P < 0.05, **P < 0.01 relative to the WT group. Differences are analyzed using Student's t-test.

Next, to further determine whether the deficiency of osteoblast-derived EGFL6 affects bone repair, 1.0-mm mono-cortical bone defects were created in 10-week-old male mice tibiae. The bone defect model recapitulates multiple stages of fracture healing, with the healing process being equivalent to that of a stabilized fracture 17, 19, 20, in which the bone repair process can be typically observed within two weeks. Thus, we chose 1-week and 2-weeks post- procedure as the time points for analysis (Figure 5A). Micro-CT scanning and histological evaluations were performed to investigate the process of bone repair. In the EGFL6-deficient mice (EGFL6^OCN^ mice), the amount of newly formed bone within the defect region was largely reduced postoperatively at week 1 when compared to that of WT control mice (EGFL6^fl/Y^ mice), as indicated by micro-CT scanning and analyses (Figure 5B-C). Additionally, whereas HE staining, picrosirius red (PSR) staining (red = collagen), and Masson Trichrome staining (blue = bone) of the injury site showed robust new bone formation and collagen accumulation in the cortical area as well as the bone marrow cavity, while the loss of EGFL6 led to a poor outcome of bone repair that featured by relatively more fibrous tissue and delayed new bone formation (Figure 5B-C). Moreover, at postoperative week 2, the EGFL6 cKO mice also exhibited a significant reduction of bone volume as examined by micro-CT and histological staining (Figure 5D-E). Thus, these data imply that EGFL6 is a crucial factor required for the process of bone repair.

### EGFL6 deficiency reduced angiogenesis and osteogenesis during bone repair

Given the indispensable role of angiogenesis in bone repair and that EGFL6 was previously reported to mediate angiogenic activity *in vitro* 7, we hypothesized that the impaired bone repair observed in EGFL6-deficient mice may be associated with vascular defects. Indeed, immunofluorescence staining of corresponding frozen sections for bone-specific type H endothelium (CD31^+^EMCN^+^) of the frozen sections indicated that the volume of type H vessels in the injury sites were largely reduced in EGFL6^OCN^ mice as compared with that in EGFL6^fl/Y^ mice (Figure 5F-G). Moreover, angiography by using micro-CT scanning and analysis also indicated that blood vessels formation in the bone injury site were decreased in EGFL6^OCN^ mice (Figure S7). Consistently, inhibiting EGFL6 using a neutralizing antibody led to the decreased amount of the vessel outgrowth from metatarsals of E17.5 embryos (Figure S8). The EGFL6 antibody was further administrated on rats following femur osteotomy procedures. Not surprisingly, angiography of femur at 6 and 9 weeks after osteotomy indicated that the rats received EGFL6 antibody have compromised angiogenesis (Figure S9A-B). X-ray and micro-CT were performed to examine the bone healing at 3 weeks, 6 weeks, and 9 weeks after operations (Figure S9C). Compared with the rats administrated with vehicle control, we found that the rats who received EGFL6 antibody displayed a bone nonunion or delayed union at the indicated time points (Figure S9D), suggesting that reduced angiogenesis is accompanied by compromised bone repair in the absence of EGFL6.

**Figure 6:**
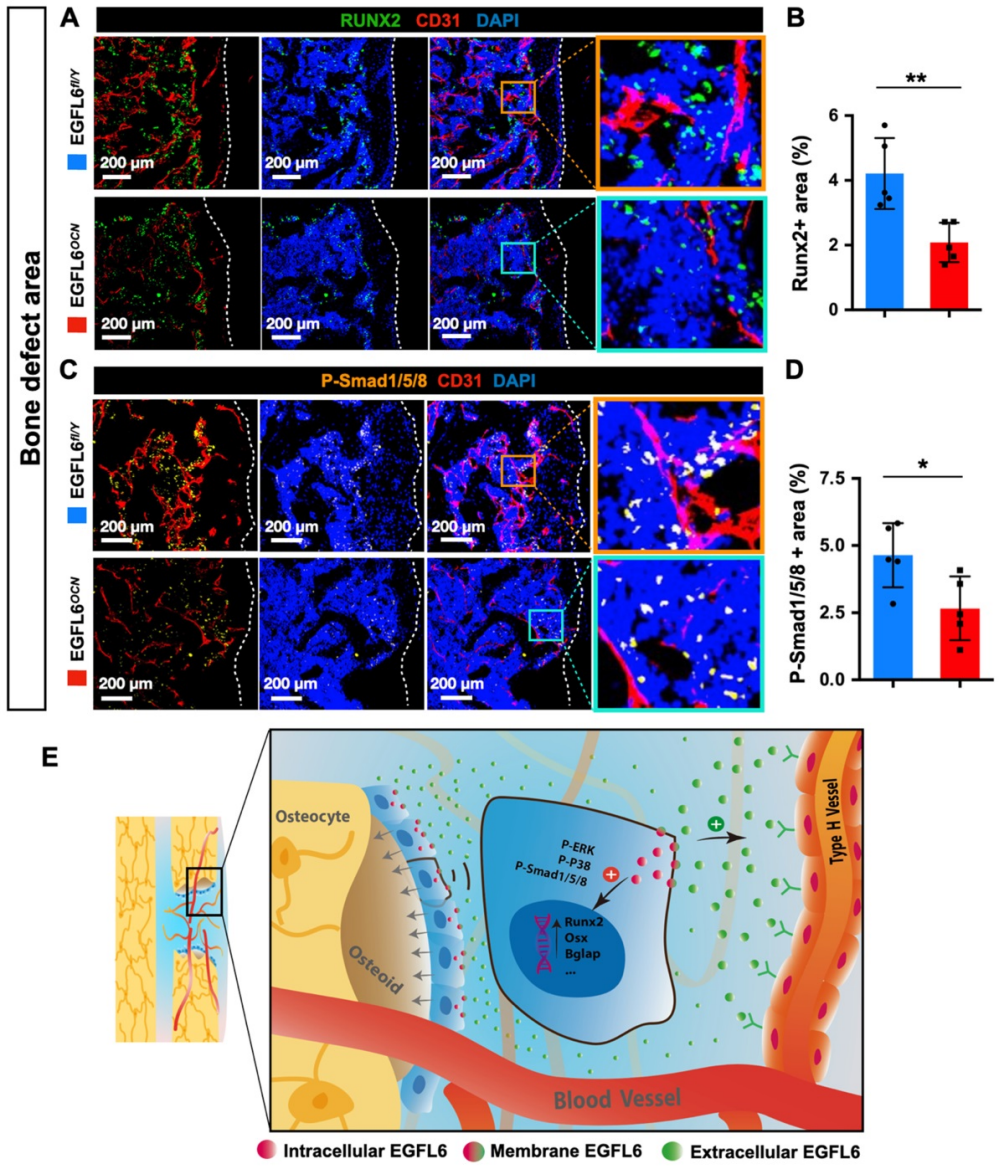
** EGFL6 deficiency reduced osteogenesis during bone repair. (A)** Representative confocal images of immunofluorescence staining for Runx2 (green) and CD31 (red) in bone defect region of EGFL6^OCN^ and EGFL6^fl/Y^ mice at week 2. **(B)** Quantification of Runx2-positive area in (A) (n = 5 per group). **(C)** Representative confocal images of immunofluorescence staining for P-Smad1/5/8 (orange) and CD31 (red) in bone defect region. **(D)** Quantification of P-Smad1/5/8-positive area in (C) (n = 5 per group). **(E)** Schematic illustration of osteoblast derived EGFL6 which contributes to the coupling of osteogenesis and angiogenesis in bone repair. White dashed lines indicate the edge of bone tissue. *P < 0.05, **P < 0.01 relative to the WT group. Differences are analyzed using Student's t-test.

Because angiogenesis and osteogenesis are tightly coupled in the context of bone, we next investigated how the lack of EGFL6 affects the osteoblast lineage cells within sites of bone injury. Immunostaining showed that the expression of Runx2, a transcription factor for osteoblast progenitors, was reduced in the bone defect region of EGFL6^OCN^ mice relative to the WT controls, suggesting the defective osteogenesis (Figure 6A-B). Mechanistically, it was determined that the attenuated BMP signaling pathway was affected due to EGFL6 insufficiency, as indicated by the immunostaining for P-smad1/5/8 (Figure 6C-D). Together, these findings together revealed that the osteoblast derived EGFL6 is essential for bone defect repair by mediating the formation of type H vessels in a paracrine manner and regulating osteoblastic osteogenesis via BMP signaling in an intracrine manner (Figure 6E).

## Discussion

Accumulated studies have highlighted the essential role of type H vessels required for bone modelling and remodeling within the bone microenvironment. In addition to acting as a passive conduit for the delivery of blood, these bone-specific endothelia tightly coupled osteogenesis by deploying a couple of angiocrine factors with pro-osteogenic capacity, such as BMPs and Noggin 5, 21. Osteoblast lineage cells are strategically located in close proximity to CD31^high^EMCN^high^ endothelium 4. Likewise, osteoblasts can secret a wide range of angiogenic factors to promote angiogenesis 22. This intimate cellular communication plays a key role in the bone physiology. However, the underlying mechanisms remain poorly understood. Previously we have reported that the osteoblast-derived angiogenic factor EGFL6 can promote the functions of endothelial cells *in vitro* and thus may serve as a promising target to bone disorders 7. In this study, we aimed to further elucidate how EGFL6 mediates osteoblast differentiation as well as investigate its contribution to bone under physiological and pathological (bone defect) conditions.

Osteoblasts are the major source of EGFL6 within the bone microenvironment, with no appreciable EGFL6 expression levels detected in either osteoclasts or endothelial cells 7. Unlike other osteoblast-derived angiogenic factors, EGFL6 is rarely expressed in other cells and tissues beyond bone. EGFL6 expression is dramatically increased during osteogenic differentiation, particularly at mid-late stage, suggesting that it may be crucial for osteoblastic function. Indeed, MSCs transduced with lentiviral EGFL6 shRNA displayed reduced bone nodule formation which was accompanied by the down-regulation of key osteogenic genes including *Bglap, Sp7,* and* Runx2*. Conversely, gain-of-EGFL6 in MSCs promoted osteoblastic mineralization as well as enhanced the expressions of osteogenic genes. Interestingly, the expression of *Vegfa* was also augmented in MSCs overexpressing EGFL6 or declined in MSCs with knocked-down EGFL6, indicating that EGFL6 may function as an upstream regulator of *Vegfa* during osteoblast differentiation. VEGF is a well-known angiogenic factor which regulates angiogenesis and osteogenesis within the bone microenvironment 17, 23. Intracellular VEGF is known to direct the differentiation of MSCs into osteoblasts rather than adipocytes by regulating Runx2 23. Therefore, EGFL6 may also enhance osteoblast differentiation indirectly by triggering the expression of VEGF.

EGFL6 has been widely reported to interact with integrin β1 in several types of cells 8-10, 24. The lack of integrin β1 in osteoblasts led to a decreased bone formation by blunting BMP2-dependent osteogenic genes 25. In the present study, EGFL6 was found to activate BMP signaling via canonical Smad-dependent and non-canonical MAPK signaling pathways. We show that osteoblasts overexpressing EGFL6 were induced with BMP2, and the phosphorylation of Smad1/5/8 was found to be significantly amplified compared with osteoblasts transduced with a control vector. In addition, our data showed that the overexpression of EGFL6 enhances BMP2 downstream events through MAPK signaling via ERK and P38. These signaling cascades converge at osteogenic transcriptional factors, Sp7 and Runx2, which promotes MSCs to differentiate into osteoblasts 26. It was previously demonstrated that the addition of exogenous soluble EGFL6 has little effect on KusaO osteoblastic cell differentiation 7, which is consistent with our finding that the recombinant EGFL6 has no effect on BMP signaling in MC3TC-E1 cells. Therefore, the osteoblast differentiation was promoted by the overexpression of EGFL6 but failed to respond to the treatment of exogenous soluble EGFL6 protein, implying that EGFL6 mediates osteoblastic function in an intracrine manner. The concept of the intracellular function of EGFL6 is reminiscent of the intracrine mechanism by which osteoblasts are regulated by intracellular VEGF but exhibit a lack of response to the extracellular VEGF 23. However, the precise intracellular targets that EGFL6 interacts with requires further investigation.

In addition to the intracrine effects of EGFL6 on osteoblasts, we reasoned that secreted EGFL6 may also regulate the angiogenesis within the bone microenvironment via paracrine actions. Given that the expression level of EGFL6 peaked at mid-late stage of osteoblast differentiation, EGFL6 was conditionally targeted in Ocn-positive osteoblast lineage cells by using cre-loxP system. Unexpectedly, mice with EGFL6-deficiency in osteoblast displayed a normal phenotype characterized by similar body length, weight, bone growth rate, trabecular and cortical bone parameters. Furthermore, to rule out the possibility that peripheral sources of EGFL6 may compensate for the lack of osteoblast-derived EGFL6 in EGFL6^OCN^ mice, EGFL6 gKO mice were also assessed in our study. Similar to the EGFL6 cKO mice, bone phenotypes and bone vascular networks remained unperturbed in EGFL6 gKO mice. However, MSCs isolated from EGFL6 gKO mice showed dramatically decreased bone formation due to the deletion of EGFL6. Together, our data together suggested that EGFL6 is a dispensable factor for the bone development and homeostasis under physiological conditions. It is plausible that the deficiency of EGFL6 may be compensated by additional angiogenic factors arising from bone or peripheral organs. Further, although bone microarticterual paramteres are unaltered in conditional and global EGFL6-deficient mice, whether bone quality or mechanical properties are affected require further investigation. Being a highly vascularized organ 27, the angiogenesis and osteogenesis in bone are well orchestrated by a wide range of factors, such as slit guidance ligand 3 (SLIT3) 16, CXCL9 18, platelet-derived growth factor type BB (PDGF-BB) 28, Notch 5, 29, hypoxia-inducible factor 1-alpha (HIF-1α) 4, 30, transforming growth factor beta (TGFβ)1 11, and fibroblast growth factor (FGF)1 11. Future work is still needed to address which of these factors, or others, may compensate for the loss of EGFL6. However, of note, we previously identified that osteoblast-expressed nephronectin (NPNT) shares the similar homology with EGFL6 and is involved in mediating angiogenesis 31, 32. In skin, NPNT is required for tissue patterning of the arrector pili muscle (APM) and the lack of NPNT leads to the upregulation of EGFL6, suggesting a potential compensatory relationship between EGFL6 and NPNT 8.

To further investigate the potential role of EGFL6 in bone, we assessed the EGFL6 cKO mice under pathological settings. Data obtained from the mono-cortical bone defect model revealed that the process of bone repair in EGFL6 cKO mice was diminished as compared with WT mice. Mice lacking EGFL6 were characterized by largely decreased post-injury new bone formation as determined by micro-CT scanning and bone morphometrical analyses. This was accompanied by a reduction of type H vessels as well as Runx2-positive osteoblast lineage cells within the bone defect area, demonstrating that EGFL6 is required by the angiogenesis and osteogenesis during bone repair process. Moreover, the declined expression of P-Smad1/5/8 during bone repair in EGFL6 cKO mice consistently suggested that EGFL6 is important for inducing BMP signaling during osteogenesis. Bone repair is known as a complex process in which the demand for angiogenesis and osteogenesis is highly increased 1, and the deficiency of EGFL6 could not be compensated by other factors in this condition. A similar phenomenon was previously reported for FGF-9, which is crucial for bone healing rather than physiological bone metabolism 19. The lack of FGF-9 or EGFL6 has little effect on basal bone turnover unless bone injury is induced. This unique and selective role of EGFL6 in the pathological process of bone repair may make it an ideal therapeutic target by causing less off-target side effects. It is also worth to mention that, in our study, EGFL6 gene expression was highly upregulated during bone repair and blockade of EGFL6 using neutralizing antibody efficiently diminished the blood vessels which was accompanied with the compromised formation of bone callus in rat femur osteotomy model. Together, these multiple lines of evidence collectively demonstrated that EGFL6 is required for bone repair.

In summary, this study has revealed, for the first time, that osteoblast-derived EGFL6 promotes osteogenic activity through the BMP-Smad and MAPK signaling pathways via an intracrine manner. In addition, EGLF6 directs angiogenesis during bone repair in a paracrine fashion. Based on these findings, we propose that EGFL6 contributes to the coupling of angiogenesis and osteogenesis, thus providing a rationale for developing EGFL6-based therapeutic strategies to ameliorate bone repair.

## Methods

### Mice

EGFL6 floxed mice (EGFL6^fl/Y^ or EGFL6^fl/fl^), in which exon 2 of EGFL6 was flanked by loxP sites using CRISPR/Cas9 system, were generated in Nanjing Biomedical Research Institute of Nanjing University. Transgenic mice expressing Cre recombinase under control of the osteocalcin promoter (OCN-Cre) were kindly provided by Professor Thomas Clemens 16. These two mouse strains were mated to generate EGFL6 conditional knock out (cKO) mice. Genomic DNA isolated from tails were routinely used for genotyping. All animals were maintained under specific pathogen-free (SPF) conditions, and all common experimental procedures were approved by the Institutional Animal Ethics Committee of Guangxi Medical University (No.201803025). The animal procedures on EGFL6 gKO mice were approved by the animal ethics committee of the University of Western Australia (RA/3/100/1539, RA3/300/111). Briefly, C57BL/6J blastocysts were injected with a mutant embryonic stem (ES) cell which has an insertion of UPA gene trap vector downstream of CMHD-GT-485H8 sequence tag in the EGFL6 genome. The blastocysts were subsequently implanted into the uterus of a female recipient mouse. Next, female mice heterozygous were generated and inter-crossed to produce EGFL6 gKO homozygous mice. Mice heterozygous for EGFL6 deletion were obtained from the Australian Phenomics Networks (Monash University, Vic, Australia).

### Cell culture

Primary murine calvarial osteoblasts were isolated from 7-day-old mice. Calvarial bone were dissected, and the soft tissues were removed. Bone was incubated in enzymatic digestion solution (serum-free a-MEM medium, 0.05% trypsin, and 1 mg/mL collagenase II) at 37 ºC in shaking warm incubator for 10 min. The supernatant of the first digestion was discarded and the subsequent 3 digestions were collected. Calvarial cells were cultured in complete a-MEM medium which contains 10% FBS and 1% penicillin/ streptomycin. Primary MSCs were isolated from 6-week-old mice by flushing the bone marrow of long bones and cultured in complete a-MEM medium 33. MC3T3-E1 cell lines were purchased from American Type Culture Collection (ATCC) and maintained in complete a-MEM medium. For osteoblast differentiation, the medium was supplemented with 50 μg/mL ascorbic acid, 5 mM β-glycerophosphate, and 10 nM dexamethasone. Full-length EGFL6 protein was purified from HEK293 using EGFL6 (NM_015507) human tagged ORF clone (RC207729) by OriGene Technologies Inc. (Rockville, US). Cells were fixed by with 2.5% glutaraldehyde solution and subjected to alkaline phosphatase (ALP) staining (BCIP/NBT liquid substrate system, Sigma) or Alizarin Red S (ARS, Sigma) staining.

### Lentivirus generation and infection

Plasmids including pLVX-Puro vector, pLVX-Puro-EGFL6, pLKO.1-Puro vector and pLKO.1-Puro containing EGFL6 shRNA sequences (#1, #2, #3) were constructed and obtained from IBSBIO (Shanghai, China). Control vectors or target constructs were co-transfected with the packaging vectors psPAX2 and pMD2G into HEK293T cells. Supernatants containing lentivirus were collected 48 h post-transfection. Lentiviruses (mixed with 5 μg/mL polybrene) were used to infect isolated primary MSCs or MC3T3-E1 cells. To select stable clones, cells were treated with 2 μg/mL puromycin until resistant cells were obtained and used for further experiments.

### Quantitative real-time PCR (qPCR) analysis

Total RNA was isolated from the cells using Trizol reagent (Life Technologies) and RNeasy Mini Kit (Qiagen). Single stranded cDNA was prepared from 1 μg of total RNA template using moloney murine leukemia virus (MMLV) reverse transcriptase with oligo-dT primer (Promega, Sydney, NSW, Australia). SYBR Green PCR MasterMix (Thermo Fisher Scientific) was used for qPCR analysis via a ViiA 7 Real-time PCR machine (Applied Biosystems). *ACTB* expression was used as a housekeeping control. The primers used for PCR are listed in Table S1.

### Western blot assay

Cells were lysed in RIPA lysis buffer on ice for protein collection. SDS-polyacrylamide gel electrophoresis (SDS-PAGE) was used to separate the protein which was subsequently transferred to a nitrocellulose membrane (GE Healthcare). Membrane was incubated in 5% skim milk for blocking, which was followed by the incubation with primary antibodies under gentle shaking overnight at 4 °C. Primary antibodies for P-Smad 1/5/8 (#9511, 1:500), Smad 1 (#9743, 1:1000), P-P38 (#4511, 1:500), P38 (#9212, 1:500), Runx2 (#12556, 1:1000) were purchased from Cell Signaling Technology. Primary antibody for osteocalcin (ab198228, 1:500) was purchased from Abcam. Primary antibodies for P-ERK (sc-7383, 1:500) and ERK (sc-514302, 1:500) were purchased from Santa Cruz Biotechnology. Primary antibody for VEGF (#MA5-13182, 1:300) was purchased from Thermo Fisher Scientific. Secondary antibodies conjugated with horseradish peroxidase (HRP) (1:3000) was incubated with the membrane on the next day. Protein bands were detected with enhanced chemiluminescence substrate (PerkinElmer), and images were captured on an Image-quant LAS 4000 (GE Healthcare).

### Micro-CT scanning and analysis

Micro-CT scanning was performed on a Skyscan 1176 micro-CT imaging system and the analysis was conducted using Bruker micro-CT software as previously described 34. Briefly, bone samples were fixed and scanned using the following parameters: resolution, 9 μm; AI 0.5 mm filter; rotation step, 0.4 degree; source voltage, 50 kV; source current, 500 μA. Volumetric images were reconstructed using NRecon software and the bone parameters were analyzed using CTAn software. For trabecular bone analysis, the volume of interest (VOI) was selected 0.5 mm above the growth plate of the distal femur and 1 mm in height (113 slices), or a height of 1mm above the end plate of the first lumbar vertebrate. Cortical bone analysis was performed in the mid shaft, 4 mm below the growth plate with a height of 1 mm (113 slices).

### Bone histomorphometry

To prepare paraffin-embedded sections, bone samples were collected and fixed in 10% neutral buffered formalin (NBF) for 24 h. Decalcification was performed by incubating the samples in 14% EDTA at 37 °C for 1 week, EDTA was changed every day. Next, bone samples were processed through ethanol series for dehydration, immersed in xylene, embedded with paraffin, and sectioned at a thickness of 5 μm. Sections were further dewaxed and stained with hematoxylin and eosin (HE), TRAP, picrosirius red (PSR), or Masson Trichrome. Images were captured using Aperio Scanscope. The number of osteoblast per bone perimeter (N.Ob/Pm), number of osteoclast per bone perimeter (N.Oc/Pm), and the parameter regarding the new bone formation in bone defect site (bone surface per tissue surface, BS/TS) were quantified using Quantitative Pathology & Bioimage Analysis (QuPath) software 35.

For immunofluorescence visualization, the cryosections were prepared and stained as previously described 36. Fresh bone tissues were fixed in ice-cold 4% paraformaldehyde (PFA) solution for 4 h. Decalcification was carried out with 0.5 M EDTA at 4 °C with constant shaking and decalcified bones were immersed into 20% sucrose and 2% polyvinylpyrrolidone (PVP) solution for cryoprotection. Finally, the tissues were embedded and frozen in 8% gelatin in the presence of 20% sucrose and 2% PVP. For immunofluorescent staining, 20-μm-thick sections were generated on a Leica microtome. Bone sections were air-dried, permeabilized in 0.3% Triton X-100, blocked in 5% BSA, and probed with the primary antibodies. The primary antibodies of CD31 (R&D, AF3628, 1:200), Endomucin (Abcam, ab106100, 1:200), EGFL6 (Abcam, ab140079, 1:100), Runx2 (Cell Signaling Technology, #12556, 1:200), P-Smad1/5/8 (#9511, 1:200, Cell Signaling Technology) were used to incubate the sections overnight at 4 °C. Sections were washed with PBS and incubated with appropriated secondary antibodies (1:500). Nuclei were counterstained with 4',6-diamidino-2- phenylindole (DAPI). Finally, sections were mounted and observed under a NIKON A1Si confocal microscope.

To assess the bone formation rates *in vivo*, 10-week-old EGFL6 WT and cKO mice were given intraperitoneal injection of calcein (25 mg/kg, Solarbio, Beijing) on day 0, followed by ARS (25 mg/kg, Solarbio, Beijing) injection on day 7. The mice were sacrificed 2 days after the second injection. Calvaria were collected and fixed in 10% NBF for 24 h and washed with PBS. The samples were embedded in paraffin and sectioned. The fluorescence was visualized and imaged using a NIKON A1Si confocal microscope. The width between the two labels was measured using NIS-Elements software and average on days to calculate mineral apposition rate (MAR).

### Bone defect model

The tibial mono-cortical bone defect mouse model was established as previously described with modifications 17, 20, 37. 10-week-old mice were used for experiments. Mice were placed under deep general anesthesia and an incision was made over the antero-proximal side of right tibia. The overlying soft tissue and muscle was divided with carefully preserving the periosteum. A 1.0 mm-diameter osseous hole that penetrated one cortex was created on the medial surface of the tibia crest using a drill. The region was irrigated with saline to remove bone dust and fragments. The muscle and skin layers were closed separately. Buprenorphine (0.05-0.1 mg/kg, s.c.) were given for analgesia after surgery.

### Statistical analysis

All values in bar graphs are presented as mean ± standard deviation (SD). The data presented in the figures represents one of at least three independent experiments. Bar graphs and statistics were prepared using GraphPad Prism 6.0 software. The differences were evaluated by two-tailed Student's t test for comparison between two groups. A P value < 0.05 was considered to be statistically significant.

## Supplementary Material

Supplementary figures, table, and methods.

## Abbreviations

ALP: alkaline phosphatase
APM: arrector pili muscle
ARS: alizarin red S
BV/TV: bone volume per tissue volume
ECs: endothelial cells
EGFL6: epidermal growth factor-like protein 6
EMCN: endomucin
FGF: fibroblast growth factor
HE: hematoxylin-eosin
HIF-1α: hypoxia-inducible factor 1-alpha
KO: knockout
MAR: mineral apposition rate
MSCs: mesenchymal stem cells
NPNT: nephronectin
bone surface per tissue surface, BS/TS): number of osteoblast per bone perimeter (N.Ob/Pm
OCN: osteocalcin
OV: overexpression
RGD: arginine-glycine-aspartic acid
PDGF-BB: platelet-derived growth factor type BB
PSR: picrosirius red
SLIT3: slit guidance ligand 3
Tb.N: trabecular number
TGFβ: transforming growth factor beta
TRAP: tartrate-resistant acid phosphatase
