# Supplementary material for: Osteoblast-derived EGFL6 couples angiogenesis to osteogenesis during bone repair
Source: Theranostics. 2021 Sep 27;11(20):9738–51. doi: 10.7150/thno.60902 (PMC8581413; doi:10.7150/thno.60902)
Supplement: Supplementary file 1 — Supplementary figures, table, and methods. [file thnov11p9738s1.pdf]

Osteoblast-derived EGFL6 couples angiogenesis to osteogenesis during bone repair

Kai Chen[1,2], Shijie Liao[1,3,4], Yicheng Li[3], Haibo Jiang[5,6], Yun Liu[1,3,4], Chao Wang[1], Vincent Kuek[1], Jacob Kenny[1], Boxiang Li[4], Qian Huang[4], Jianxin Hong[7], Yan Huang[8], Shek Man Chim[1], Jennifer Tickner[1], Nathan Pavlos[1], Jinmin Zhao[3,4], Qian Liu[1, 3], An Qin[8*], Jiake Xu[1 *]

1. School of Biomedical Sciences, University of Western Australia, Perth, WA, Australia.
2. Department of Orthopedics, The First Affiliated Hospital of Wenzhou Medical University, Wenzhou, Zhejiang, China
3. Guangxi Key Laboratory of Regenerative Medicine, Guangxi Medical University, Nanning, China.
4. Department of Orthopedics, The First Affiliated Hospital of Guangxi Medical University, Nanning, Guangxi, China
5. School of Molecular Sciences, University of Western Austra›lia, Perth, WA, Australia.
6. Department of Chemistry, The University of Hong Kong, Pokfulam Road, Hong Kong, P. R. China
7. Wenzhou Medical University, Ouhai District, Wenzhou, Zhejiang, China
8. Shanghai Key Laboratory of Orthopedic Implants, Department of Orthopaedics , Shanghai Ninth People's Hospital, Shanghai Jiao Tong University School of Medicine, Shanghai, China.

*Corresponding authors: 1. Prof. Jiake Xu, jiake.xu@uwa.edu.au
2. Dr. An Qin, dr.qinan@163.com

Supplementary Figures

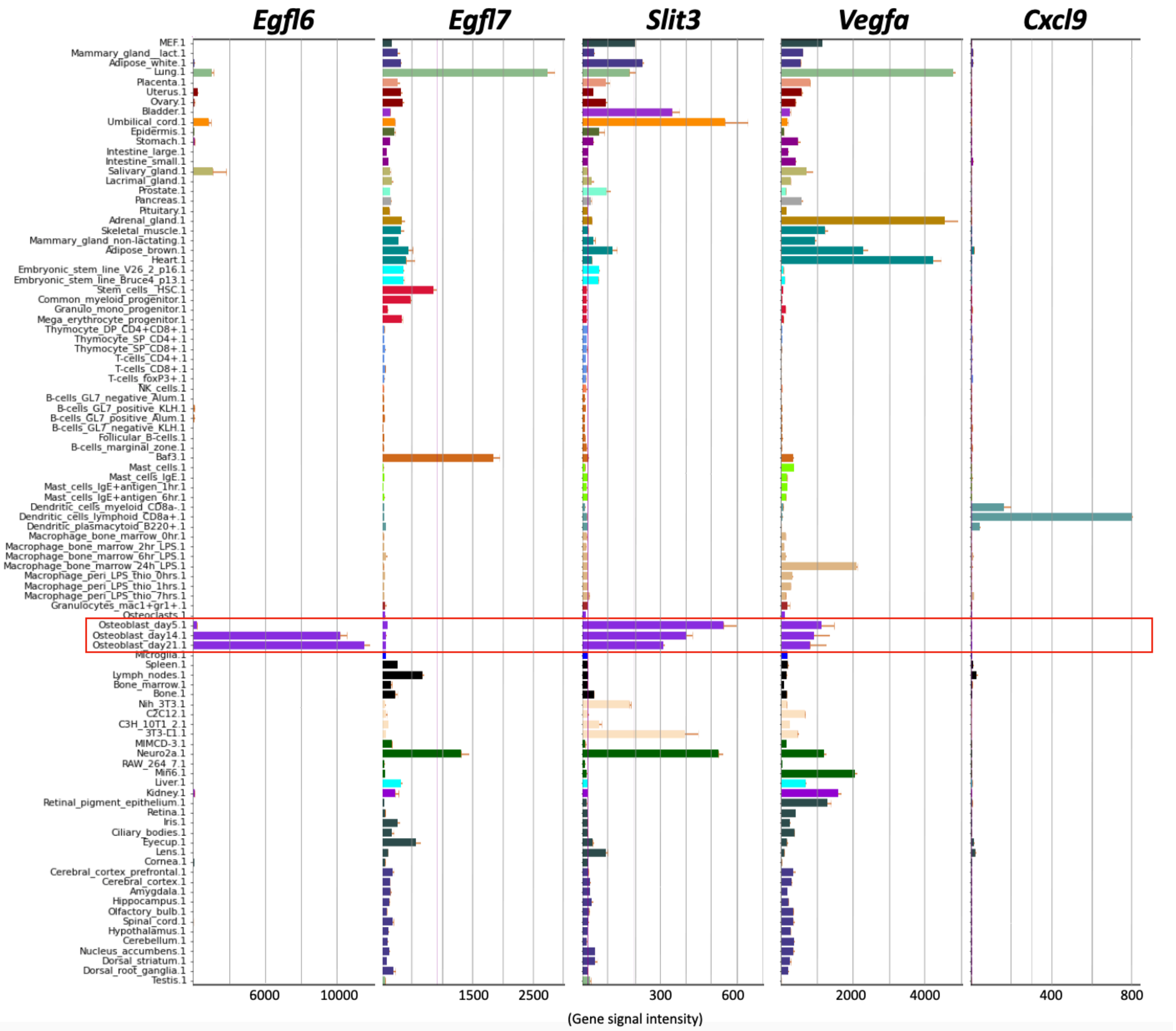

Figure S1 The expression levels of several osteoblast-derived angiogenic factors profiled from an array of normal tissues, organs, and cell lines in mice. Data is adapted from BioGPS (http://biogps.org/).

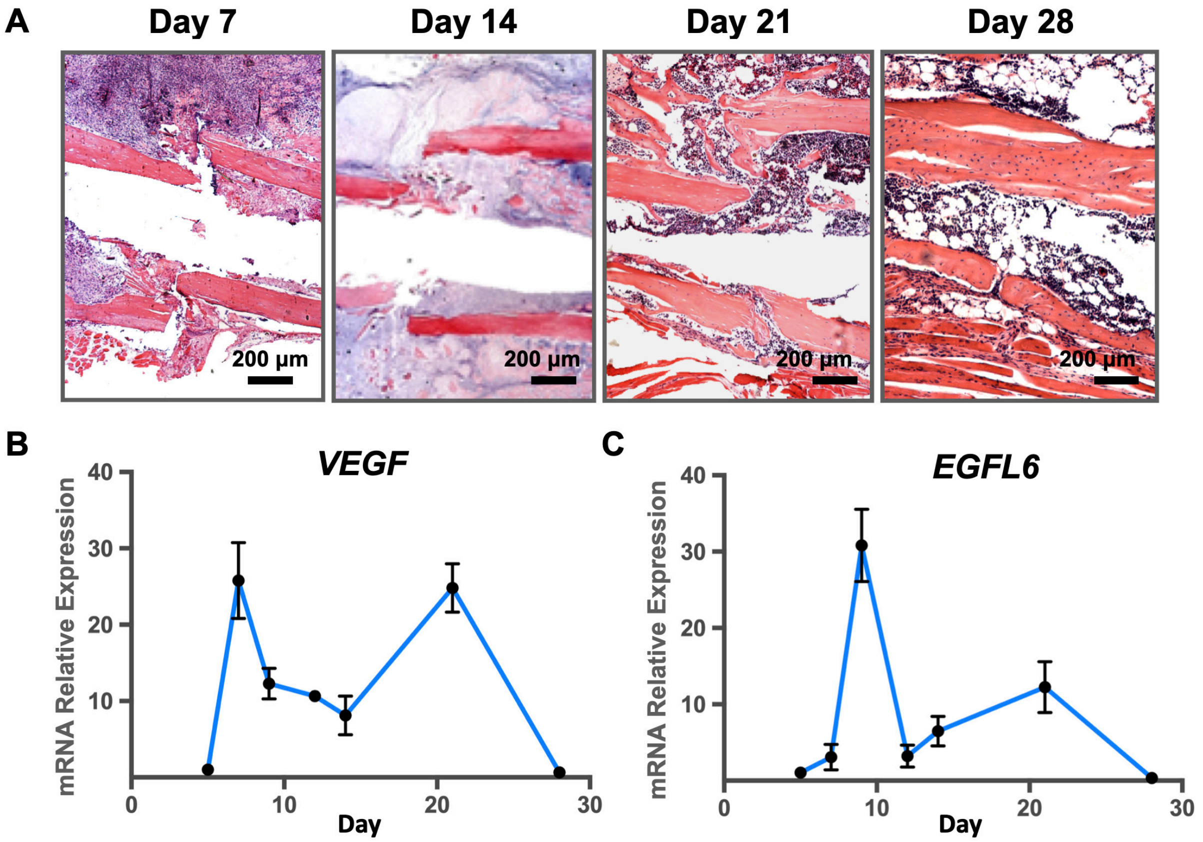

Figure S2 The expression level of VEGF and EGFL6 genes in osteotomy healing model. **(a)** Representative hematoxylin-eosin (HE) staining of tibia healing at day 7, 14, 21, 28 in 8-week-old mice. **(b)** qPCR analysis of the mRNA expression of VEGF and EGFL6 in bone calluses at day 5, 7, 9, 12, 14, 21, and 28 after fracture (n = 8). All data are presented as mean ± SD.

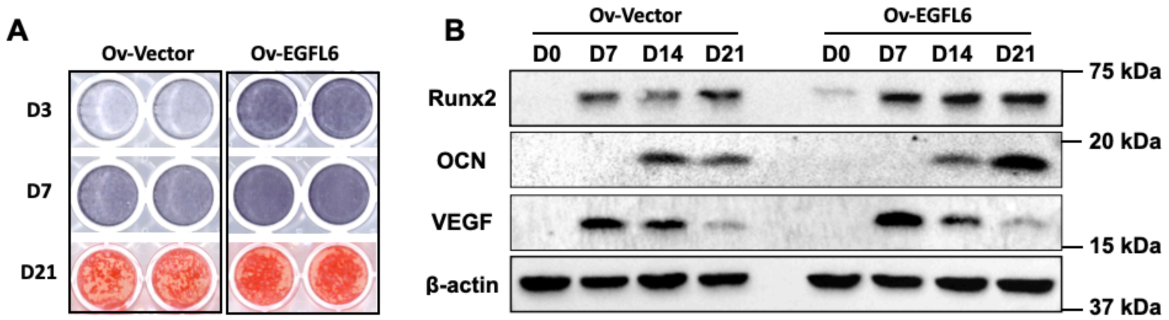

Figure S3. Overexpression of EGFL6 promotes osteogenesis. (a) Alkaline phosphatase (ALP) and Alizarin Red S (ARS) staining of osteogenic differentiation of MC3T3-E1 cells with or without EGFL6 overexpression. **(b)** Western Blot assay showing the protein expressions of Runx2, osteocalcin (OCN), and vascular endothelial growth factor (VEGF) during osteoblast differentiation and mineralization.

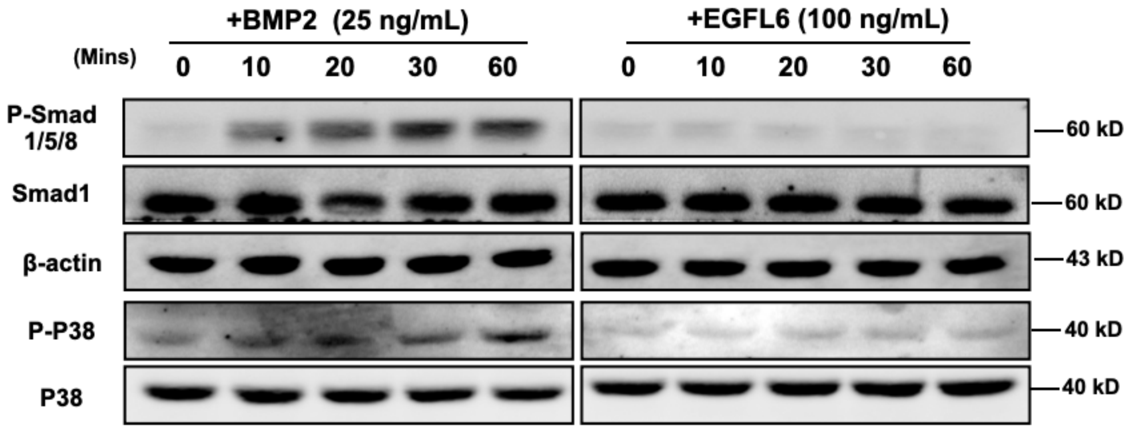

Figure S4. The addition of exogenous EGFL6 protein fails to activate BMP signaling in osteoblasts. MC3T3-E1 cells were induced by the BMP2 (25 ng/mL) or EGFL6 protein (100 ng/mL) for the time course as indicated. The proteins level of BMP signaling pathway including P-Smad 1/5/8, P-ERK, and P-P38 were examined using Western Blot assay.

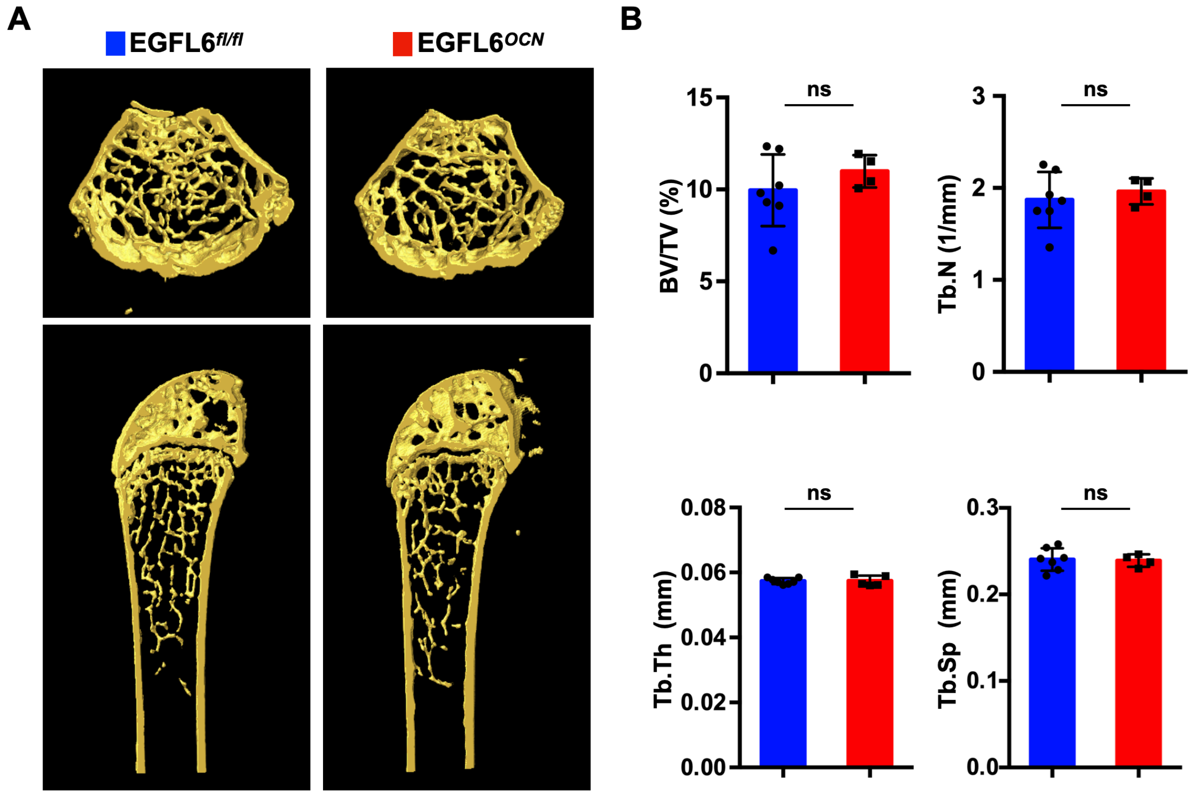

Figure S5. Osteoblast-specific deletion of EGFL6 in female mice has no little effect on bone phenotype. (a) Representative three-dimensional reconstructed micro-CT images showing the femurs of 12-week-old female EGFL6^{OCN} and EGFL6$^{fl/fl}$ mice. **(b)** Quantification of the trabecular bone parameters including bone volume per tissue volume (BV/TV), trabecular number (Tb.N), trabecular thickness (Tb.Th), and trabecular separation (Tb.Sp) (EGFL6$^{fl/fl}$ mice, n = 7; EGFL6^{OCN} , n = 4). All bar graphs are presented as mean ± SD. ns, no significance. Differences are analyzed using Student's t-test.

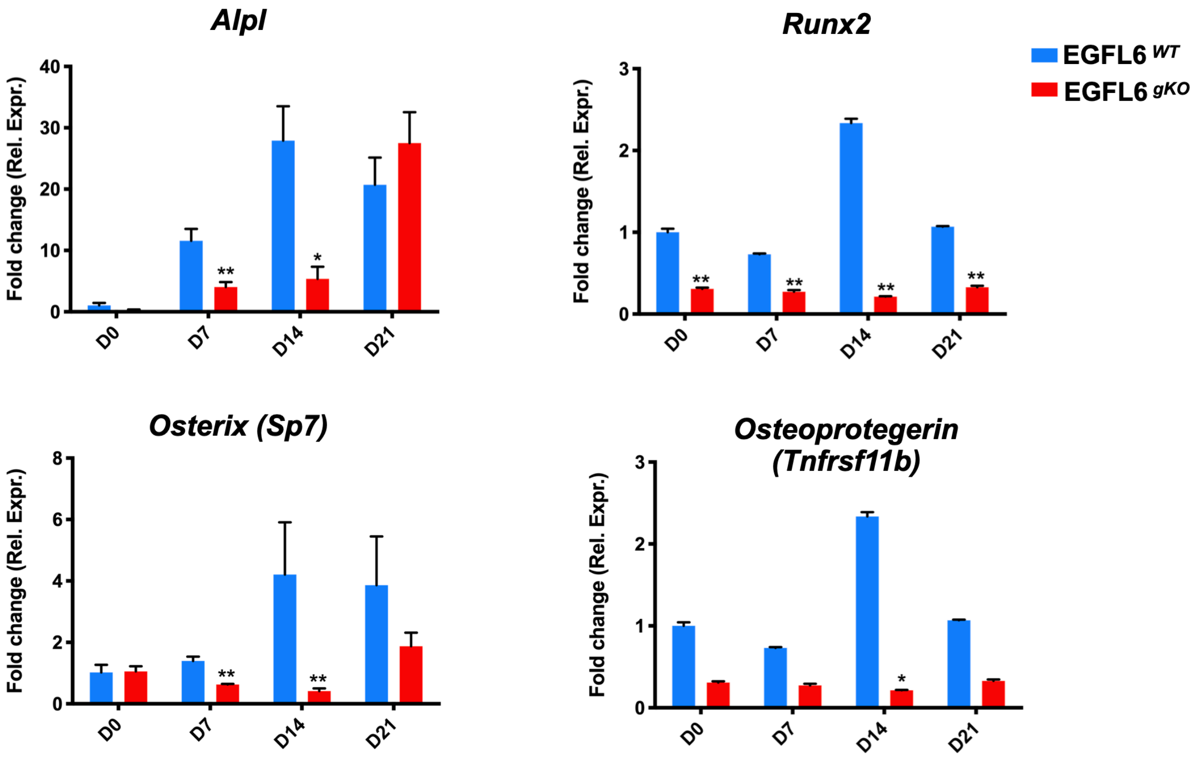

Figure S6 Bone marrow mesenchymal stem cells (MSCs) derived from EGFL6^{gKO} mice exhibit down-regulated expression of osteoblast-related genes.

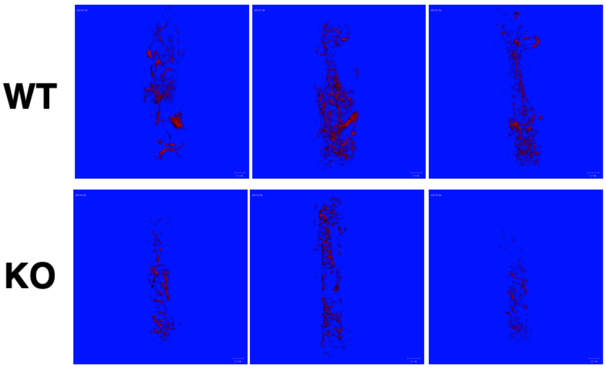

	Vascular volume fraction (%)		
WT	0.57	0.34	0.24
KO	0.29	0.27	0.17

Figure S7 Osteoblast-specific deletion of EGFL6 inhibits blood vessel formation in bone injury sites. Angiography by using micro-CT scanning and analysis of formation of blood vessels in three pairs of mice osteotomy models (six weeks after procedure).

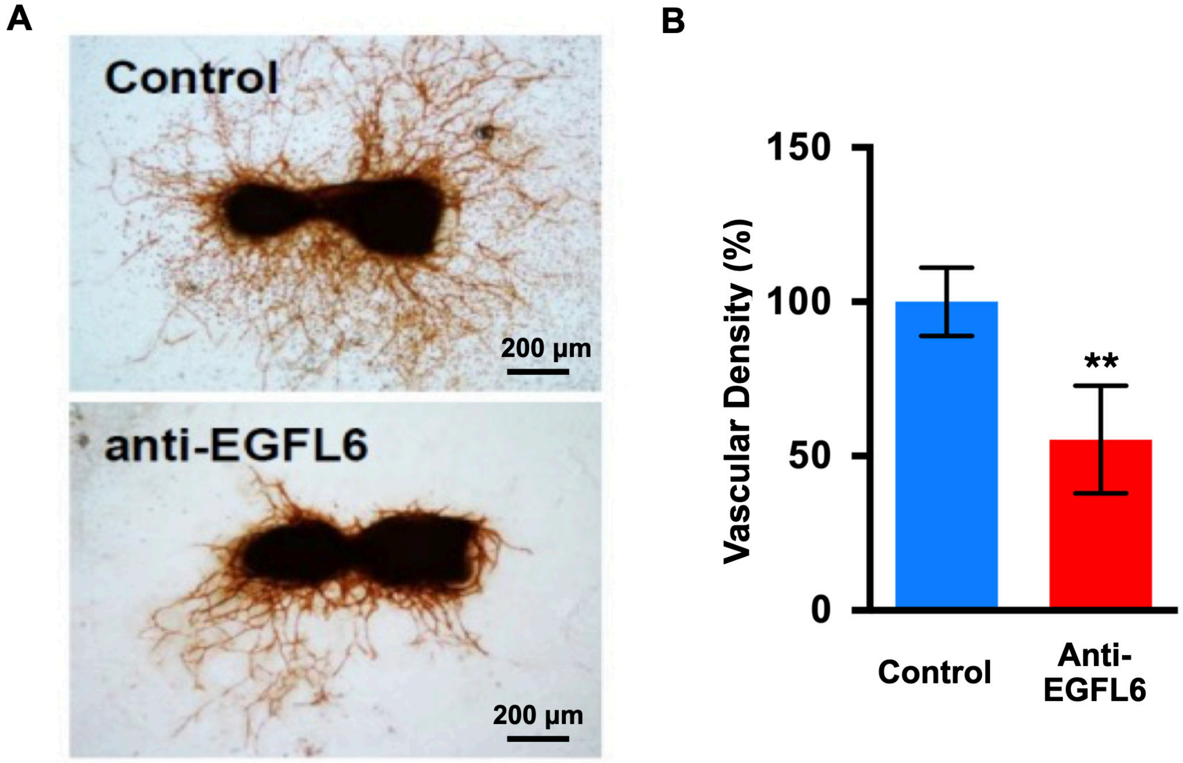

Figure S8 The inhibition of EGFL6 reduces the angiogenesis of metatarsals. **(a)** Representative images showing the inhibition of EGFL6 using EGFL6 antibody suppressed the vessel outgrowth from metatarsals of E17.5 embryos. **(b)** Quantitative analysis of vessel density (n = 4 per group). **P < 0.01 relative to the control group. Differences are analyzed using Student’s t-test.

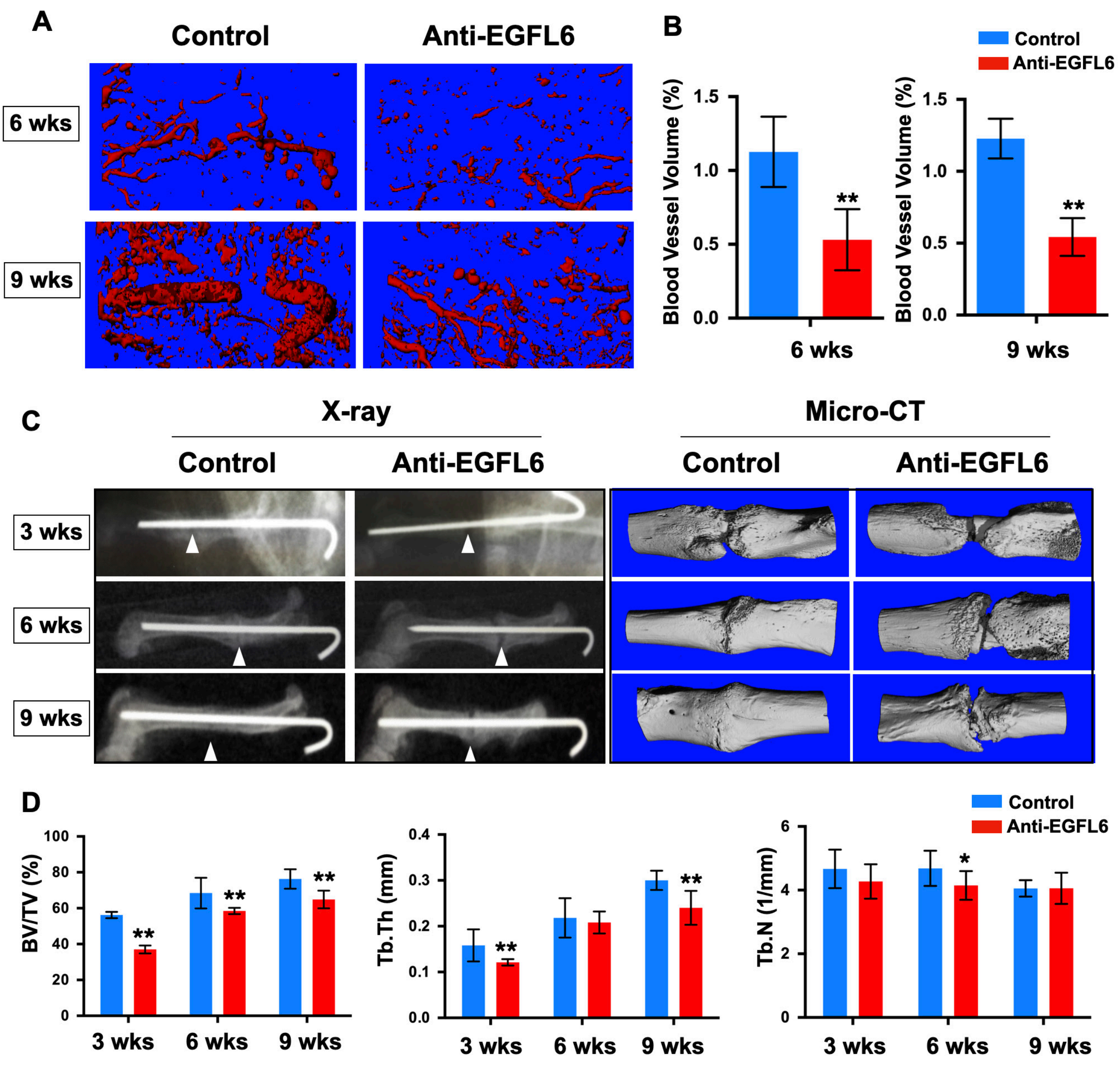

Figure S9 The inhibition of EGFL6 leads to impaired angiogenesis accompanied with delayed bone fracture union. (a) Representative angiography of femur at 6 and 9 weeks after fracture in rats receiving vehicle or EGFL6 antibody. **(b)** Quantification of blood vessel volume in (c) (n = 3 per group). **(c)** Representative X-ray and micro-CT images of rat femur fracture healing at 3, 6, 9 weeks after surgical procedure in mice receiving vehicle or EGFL6 antibody. **(d)** Quantification of the fracture callus of (a) (n = 3 per group). BV/TV, bone volume per tissue volume; Tb.N, trabecular number; Tb.Th, trabecular thickness. All data are presented as mean ± SD. *P < 0.05, **P < 0.01 relative to the WT group. Differences are analyzed using Student's t-test.

Table S1. Primer Sequences

Genes	Forward Sequence (5'-3')	Tm (°C)	Amplicon Size
EGFL6 (NM_019397.3)	Forward (5'-3'): AGCCTGGGGTCTGTCAGTAT	59.96	97 bp
	Reverse (5'-3'): TGGGCTCACACATAGCTTCG	60.11	
Bglap (NM_007541.3)	Forward (5'-3'): GCGCTCTGTCTCTCTGACCT	61.31	89 bp
	Reverse (5'-3'): ACCTTATTGCCCTCCTGCTT	59.00	
Sp7 (NM_130458.4)	Forward (5'-3'): GTCCTCTCTGCTTGAGGAAGAA	59.44	155 bp
	Reverse (5'-3'): GGGCTGAAAGGTCAGCGTAT	60.11	
Runx2 (NM_001146038.2)	Forward (5'-3'): CTTCGTCAGCATCCTATCAGTTC	58.95	145 bp
	Reverse (5'-3'): TCAGCGTCAACACCATCATTC	58.92	
Vegfa (NM_001025257.3)	Forward (5'-3'): ATCTTCAAGCCGTCCTGTGTG	63.00	102 bp
	Reverse (5'-3'): ATCTTCAAGCCGTCCTGTGTG	59.96	
Actb (NM_007393.5)	Forward (5'-3'): AAGATCAAGATCATTGCTCCTCCT	59.59	171 bp
	Reverse (5'-3'): AGCTCAGTAACAGTCCGCCT	60.90	
Tnfrsf11b (NM_008764.3)	Forward (5'-3'): GCCACGCAAAAGTGTGGAAT	59.97	123 bp
	Reverse (5'-3'): TTTGGTCCCAGGCAAACTGT	60.03	
Alpl (NM_007431.3)	Forward (5'-3'): CAGGCCGCCTTCATAAGCA	60.45	132 bp
	Reverse (5'-3'): AATTGACGTTCCGATCCTGC	58.63	

Supplementary Methods

Fetal metatarsal angiogenesis assay

The fetal metatarsal angiogenesis assay was carried out as previously described [1, 2] and the experimental procedures were approved by the approved by the University of Western Australia Animal Ethics Committee. In brief, metatarsals were aseptically dissected from E17.5 embryos and cultured in complete α-MEM medium for 72 h. Next, the medium was replaced with fresh medium in the presence or absence of EGFL6 antibody for 2 weeks prior to fixation and staining for CD31 (Abcam). Polyclonal anti-GST-EGFL6 was generated and identified as we previously reported [3]. Polyclonal anti-GST was used as a control group. The images were acquired under a Nikon Eclipse TE2000-5 microscope and the tube-like structures were measured quantitatively using Image J software.

Animal osteotomy models

The following experimental procedures in mice were performed according to the policies of Shanghai Jiao Tong University and approved by the Institutional Animal Ethics Committee. The experimental procedures in rats were performed and approved in Animal Experimental Center of Zhejiang Taizhou Hospital. To investigate the expression pattern of EGFL6 during

bone healing, osteotomy models were created as previously described [4]. Eight-week-old mice were placed under anesthesia by intramuscularly injecting pentobarbital sodium (0.05 mg/g). Under aseptic conditions, a tibiae fracture was created in each mouse by sawing the shaft and a pin was inserted for intramedullary fixation. At time points as indicated, tibia was harvested for histology, or the bone callus was harvested for RNA extraction and qPCR analysis. To determine whether the blocking of EGFL6 affect bone healing, we established rat osteotomy models as previously described with modifications [5, 6], which was followed by the treatment with EGFL6 antibody. In brief, 4-month-old rats were anesthetized by the injection of sodium pentobarbital. Next, the shaft of femur was exposed, and osteotomy was created by using an oscillating saw. A 1.2-mm diameter Kirschner wire was inserted into the femoral canal to achieve stability. Vehicle or EGFL6 antibody (100 ul per rat, twice a week) were injected into fracture sites. Femur was harvested at time points including 3, 6, and 9 weeks for X-ray, micro-CT scanning or micro-angiography.

Micro-CT based angiography

Micro-CT based angiography was carried out according to a previous study [7] with modifications. Rats or mice were placed under anesthesia and subjected to a thoracotomy which was followed by heparinized perfusion through the circulatory system via the left cardiac ventricle. After the draining of blood flow, rats were proceeded to vessel fixation by the perfusion of 10% neutral buffered formalin (NBF). Subsequently, contrast agent (Microfil) was infused thoroughly. Thereafter, animals were kept at 4℃ overnight prior to samples collection and micro-CT scanning.

References

1. Deckers M, van der Pluijm G, Dooijewaard S, Kroon M, van Hinsbergh V, Papapoulos S, et al. Effect of angiogenic and antiangiogenic compounds on the outgrowth of capillary structures from fetal mouse bone explants. Lab Invest. 2001; 81: 5-15.
2. Chim SM, Kuek V, Chow ST, Lim BS, Tickner J, Zhao J, et al. EGFL7 is expressed in bone microenvironment and promotes angiogenesis via ERK, STAT3, and integrin signaling cascades. J Cell Physiol. 2015; 230: 82-94.
3. Chim SM, Qin A, Tickner J, Pavlos N, Davey T, Wang H, et al. EGFL6 promotes endothelial cell migration and angiogenesis through the activation of extracellular signal-regulated kinase. J Biol Chem. 2011; 286: 22035-46.
4. Huang Y, Zhang X, Du K, Yang F, Shi Y, Huang J, et al. Inhibition of beta-catenin signaling in chondrocytes induces delayed fracture healing in mice. J Orthop Res. 2012; 30: 304-10.

5. Mishima K, Kitoh H, Ohkawara B, Okuno T, Ito M, Masuda A, et al. Lansoprazole Upregulates Polyubiquitination of the TNF Receptor-Associated Factor 6 and Facilitates Runx2-mediated Osteoblastogenesis. EBioMedicine. 2015; 2: 2046-61.
6. Suen PK, He YX, Chow DH, Huang L, Li C, Ke HZ, et al. Sclerostin monoclonal antibody enhanced bone fracture healing in an open osteotomy model in rats. J Orthop Res. 2014; 32: 997-1005.
7. He YX, Liu Z, Pan XH, Tang T, Guo BS, Zheng LZ, et al. Deletion of estrogen receptor beta accelerates early stage of bone healing in a mouse osteotomy model. Osteoporos Int. 2012; 23: 377-89.
